# Fructose, Glucocorticoids and Adipose Tissue: Implications for the Metabolic Syndrome

**DOI:** 10.3390/nu9050426

**Published:** 2017-04-26

**Authors:** Balázs Legeza, Paola Marcolongo, Alessandra Gamberucci, Viola Varga, Gábor Bánhegyi, Angiolo Benedetti, Alex Odermatt

**Affiliations:** 1 Division of Molecular and Systems Toxicology, Department of Pharmaceutical Sciences, University of Basel, Klingelbergstrasse 50, 4056 Basel, Switzerland; balazs.legeza@dkf.unibe.ch; 2 Department of Medical Chemistry, Molecular Biology and Pathobiochemistry, Semmelweis University, Budapest 1085, Hungary; varviola@gmail.com (V.V.); banhegyi.gabor@med.semmelweis-univ.hu (G.B.); 3 First Department of Pediatrics, Semmelweis University, Budapest 1085, Hungary; 4 Department of Molecular and Developmental Medicine, University of Siena, 53100 Siena, Italy; paola.marcolongo@unisi.it (P.M.); alessandra.gamberucci@unisi.it (A.G.); benedetti@unisi.it (A.B.); 5 Pathobiochemistry Research Group of the Hungarian Academy of Sciences and Semmelweis University, Budapest 1085, Hungary

**Keywords:** fructose, glucocorticoid, obesity, metabolic syndrome, adipogenesis

## Abstract

The modern Western society lifestyle is characterized by a hyperenergetic, high sugar containing food intake. Sugar intake increased dramatically during the last few decades, due to the excessive consumption of high-sugar drinks and high-fructose corn syrup. Current evidence suggests that high fructose intake when combined with overeating and adiposity promotes adverse metabolic health effects including dyslipidemia, insulin resistance, type II diabetes, and inflammation. Similarly, elevated glucocorticoid levels, especially the enhanced generation of active glucocorticoids in the adipose tissue due to increased 11β-hydroxysteroid dehydrogenase 1 (11β-HSD1) activity, have been associated with metabolic diseases. Moreover, recent evidence suggests that fructose stimulates the 11β-HSD1-mediated glucocorticoid activation by enhancing the availability of its cofactor NADPH. In adipocytes, fructose was found to stimulate 11β-HSD1 expression and activity, thereby promoting the adipogenic effects of glucocorticoids. This article aims to highlight the interconnections between overwhelmed fructose metabolism, intracellular glucocorticoid activation in adipose tissue, and their metabolic effects on the progression of the metabolic syndrome.

## 1. Introduction

Our ancestors obtained their food from hunting and gathering, but the transition to modern Western society lifestyle with its tremendous technological advances in food processing led to extensive changes in food intake and composition. The Western-style diet, also called the meat-sweet diet is characterized by high intakes of processed foods rich in saturated fat, trans-fatty acids, proteins from red meat, and sodium, as well as an excessive consumption of sugar [1]. In line with this transition, obesity has emerged as a major global health problem in the last few decades [2]. Epidemiologic studies pointed out that overweight and obesity are important risk factors of type II diabetes mellitus (T2DM) and cardiovascular disease (CVD) [3,4,5,6]. The involvement of adiposity—predominantly splanchnic obesity—in the development of the metabolic syndrome (MetS) has been well established [7]. Metabolic and endocrine factors, like hormones and para/autocrine mediators, have been shown to stimulate adipocyte proliferation and differentiation [8]. When the adipose tissue reaches a critical mass and/or hypoxia occurs, a cellular signaling response triggers a switch from oxidative metabolism to anaerobic glycolysis and increases the secretion of a number of inflammation-related adipokines accompanied with cell damage [9]. On the other hand, this inflammatory response is also a key factor for modulating insulin sensitivity in adipose tissue and the development of obesity-associated diseases [10]. The chronic low-grade inflammation is a characteristic of obesity, whereby adipose tissue releases many inflammatory mediators, including TNF-α, IL-1β, and IL-6, and plasma concentrations of these secreted pro-inflammatory cytokines were found to be elevated in obese individuals [11].

The rate of dietary fructose consumption, mostly in combination with glucose, continued to rise worldwide over the last fifty years [12,13], and numerous human and animal studies demonstrated a link to the rising prevalence of obesity, T2DM, and MetS [14,15,16,17]. Fructose, which is found in fruits, became a major component of the modern diet by robust intake of sucrose (table sugar, consisting of one molecule of glucose and one molecule of fructose and subjected to cleavage in the intestinal tract) and synthetic high fructose corn syrup (HFCS, consisting of a mixture of glucose and fructose with a ratio close to one) [18] that is currently added to beverages and foods. Compared with glucose, fructose has a lower glycemic index, does not generate an insulin response, but has a slightly higher sweetening power. Furthermore, fructose is a potent lipogenic and adipogenic nutrient. For example, fructose rich diet intake increased the adipogenic potential on adipocyte precursor cells (APCs) and hence accelerated adipocyte hypertrophy [19].

Besides dietary fructose, an increased intracellular glucocorticoid production, especially in adipose tissue, has also been suggested to contribute to the pathogenesis of the MetS [20,21,22], and evidence was provided for a possible link between fructose and glucocorticoid activation [23,24,25]. The circulating and locally produced glucocorticoids have a crucial role in modulating adipocyte function as well as proliferation/differentiation [26,27]. The intracellular active glucocorticoids (cortisol and corticosterone are the major glucocorticoids in humans and rodents, respectively) are generated from their inert forms (cortisone and 11-dehydrocorticosterone) by the 11-oxoreductase activity of 11β-hydroxysteroid dehydrogenase type 1 (11β-HSD1) [28,29], a luminally oriented enzyme of the endoplasmic reticulum (ER) membrane [30,31], which is abundantly expressed in liver and adipose tissue [32,33,34].

In the adipocyte, fructose metabolism results in the generation of precursors of fatty acid synthesis and induces NADPH-generating enzymes. Recent observations indicated that fructose increases the expression of its transporter GLUT5 in the adipocyte plasma membrane and of 11β-HSD1 [35], thereby further enhancing the capability to generate active glucocorticoids in adipose tissue [25].

The purpose of the present work was to highlight the multifaceted connections between fructose metabolism and the production of active glucocorticoids in the adipose tissue and its impact on the development and progression of MetS.

## 2. Dietary Fructose and Adiposity

Epidemiological studies have linked dietary fructose consumption, either in the form of sucrose or HFCS, with an increased rate of co-occurring diseases of the MetS, such as CVD, T2DM, and non-alcoholic fatty liver disease (NAFLD) [36,37,38,39]. A cross-sectional study among adults from the National Health and Nutrition Examination study (NHANES) 1999–2006 found an association between the consumption of dietary added sugars, as assessed by 24 h dietary recall, and blood lipid measures, with significant increases in mean triglyceride (TG) levels and decreases in high-density lipoprotein (HDL)-cholesterol levels [36]. This study including more than 6000 adults, did not distinguish between fructose and glucose consumption but investigated associations with total dietary sugar consumption. Another study, using econometric models of repeated cross-sectional data on diabetes and nutritional components of food, reported on an association of a high sugar intake with T2DM, an effect that was modified but not confounded by overweight or obesity and that was not dependent on a sedentary lifestyle [37]. Furthermore, an analysis of dietary history and paired serum and liver tissue from patients with NAFLD and gender, age, and body mass index matched controls revealed a 2–3 fold higher fructose consumption (in the form of HFCS) in NAFLD patients with increased hepatic fructokinase (ketohexokinase, KHK) and fatty acid synthase expression, indicating elevated lipogenesis [38]. Dietary information was prospectively collected. Especially the consumption of sugar sweetened beverages have been regarded as critical for development of obesity, hypertension, and T2DM [14,40,41,42,43]. Another recent observational clinical study revealed increased visceral adipose tissue (VAT) using computer tomography in 1003 participants in response to sugar-sweetened beverages [44]. In this large, prospective cohort study the participants were categorized according to sugar-sweetened beverage intake frequency (non-consumers; <1 serving/week; <1 serving/day and daily consumers) and examined the adverse changes in quality and quantity of VAT after a period of six years. The study concluded that fructose, as the main component, may trigger insulin resistance and increased fat accumulation in VAT found in consumers of excessive amounts of sugar-sweetened beverages. However, it remains entirely uncertain whether the fructose or the glucose component of the sweetener or both are responsible for the metabolic effects associated with sugar-sweetened beverage consumption. Also, the pathophysiological mechanisms involved in the process contributing to the increased risk of T2DM and CVD remain to be elucidated. 

An earlier clinical study evaluated the relative effects of the consumption of glucose- and fructose-sweetened beverages in overweight and obese individuals, where these beverages covered 25% of the total energy requirements for 10 weeks [45]. Although both sweetener beverages exhibited similar weight gain, the results showed that consumption of fructose-sweetened but not glucose-sweetened beverages increased de novo lipogenesis, specifically promoting lipid deposition in VAT, stimulating dyslipidemia, altering lipoprotein remodeling and decreasing insulin sensitivity in overweight/obese adults [45]. It is still uncertain whether the observed effects of this study are comparable with the results when pure fructose-sweetened beverages are consumed, or when isocaloric fructose and/or glucose are combined with different fat diets. A critical issue remains that fructose consumption and obesity are linked and that so far no clear association between fructose intake and cardiometabolic disease has been demonstrated conclusively in the absence of overeating and weight gain [40,46,47,48].

According to another concept, fructose could contribute to obesity by stimulating sterol receptor element binding protein 1c (SREBP-1c) independently of insulin, which activates genes involved in de novo lipogenesis [49], generating fatty acids for TG production in the liver. An increase in fasting plasma TG has been observed upon excessive dietary fructose ingestion in healthy individuals as well as patients with T2DM [50,51]. Furthermore, increased hepatic lipid levels are associated with increased very low density lipoprotein (VLDL) synthesis and secretion. An elevation of systemic free fatty acid and VLDL results in increased lipid uptake in peripheral organs, such as adipose tissue and skeletal muscle, contributing to the systemic insulin resistance [15]. Additionally, fructose was proposed to promote leptin resistance, worsening obesity and insulin resistance [52]. Insulin resistance may be a secondary cause of obesity upon consumption of a hyperenergetic and high fructose-containing diet [53,54], again pointing to the importance of dissecting the direct impact of fructose and the consequences of overeating and obesity. Thus, further studies are needed to address the effects of fructose and sucrose intake under isocaloric dietary regimens and in defined subgroups, including patients with obesity, T2DM, and CVD.

An obesogenic effect of large doses of fructose was also observed in animal studies. Rats fed a diet consisting of 60% of energy derived from fructose [55], and rhesus monkeys on a daily fructose intake of 30% of ingested calories [56] had increased adipose tissue weight and several other features of MetS [57]. The fructose fed monkeys, besides adiposity, also displayed dyslipidemia, insulin resistance, and enhanced inflammatory mediators. While existing evidence convincingly shows that markers of MetS including TG, dyslipidemia, insulin resistance, and inflammatory mediators are enhanced upon high fructose containing diets [46,47,48], no consistent effect of fructose on markers of MetS could be found in studies using a defined weight-maintenance diet [47], suggesting that high fructose ingestion combined with overeating and adiposity may be responsible for the adverse health effects. 

## 3. Fructose Metabolism in Adipose Tissue

**Figure 1:**
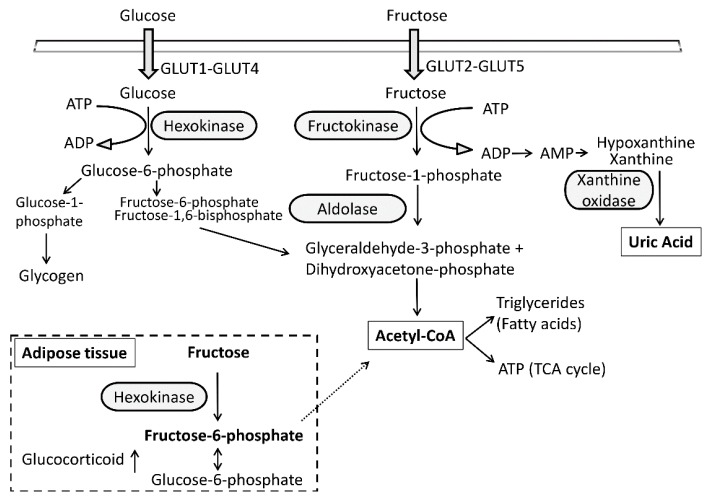
Intracellular metabolism of fructose and glucose. The intracellular metabolism of fructose differs from that of glucose primarily due to its different transporters and initial enzymatic steps. The main fructose metabolizing enzyme is fructokinase (ketohexokinase, KHK), which uses ATP to phosphorylate fructose to fructose-1-phosphate. Since this reaction is poorly regulated, the administration of excessive fructose results in rapid depletion of intracellular ATP levels, activation of AMP deaminase, and generation of uric acid. In adipocytes due to the lack of fructokinase, fructose is metabolized by hexokinase to fructose-6-phosphate, which can be converted to glucose-6-phosphate that can promote the intracellular production of glucocorticoids via stimulation of 11β-HSD1 activity.

Upon intestinal absorption, fructose is primarily metabolized by KHK to fructose-1-phosphate in the liver, which is subsequently converted to triose phosphates and in this form can supply glycolysis, lipid synthesis, gluconeogenesis, and/or glycogenesis pathways [58]. Fructose metabolism to triose phosphates differs from that of glucose: it occurs independently of insulin and without the negative feedback regulation of phosphofructokinase in the glycolytic pathway. The excessive consumption of fructose challenges the capacity of the liver, and to a lesser extent fructose remains in the systemic circulation, resulting in its utilization in peripheral tissues. Although adipocytes express GLUT5 [59] and are able to take up fructose [60], the functional role of this fructose transporter in the adipose tissue is not fully understood. In this regard, a novel significance of fructose and GLUT5 was pointed out in regulating adipocyte differentiation [61]. Furthermore, it is known that the pathway of fructose utilization in adipose tissue is largely different from that of the hepatic metabolism (Figure 1). In contrast to hepatocytes, adipocytes lack KHK and are equipped with hexokinase, which catalyzes the phosphorylation of fructose to fructose-6-phosphate (F6P) [62]. This obligatory intermediate, F6P, can be converted to glucose-6-phosphate (G6P) by G6P isomerase in the cytoplasm and the ER [25], or can be further metabolized by intermediary metabolic pathways. Investigating these roads and predicting the metabolic responses of adipocytes to fructose is challenging.

An important experimental model represents the use of ^13^C labeled fructose for in vitro and in vivo investigations. A recent study was performed using a stable isotope based dynamic profiling (SIDMAP) method with labeled [U-^13^C_6_]-D-fructose in differentiating and differentiated adipocytes (Simpson-Golabi-Behmel Syndrome (SGBS) adipose cells) exposed with an escalating range of fructose equivalent to predict the metabolic responses in detail [63]. Varma and colleagues found that increasing concentrations of fructose triggered the pyruvate conversion to acetyl-CoA via the pyruvate dehydrogenase reaction to form glutamate. The pyruvate dehydrogenase flux derived increased entry into the TCA cycle also resulted in an expanded acetyl-CoA/citrate cycling into fatty acid synthesis and free palmitate release [63]. These results explored the lipogenic potential of fructose in adipocytes and how fructose acts as an anabolic substrate for molecular synthesis and energy storage and much less so for oxidation. On the other hand, when the intermediary metabolism of glucose was investigated with the same method using [1,2-^13^C_2_]-d-glucose under the influence of increasing concentrations of fructose, their data showed that fructose dose-dependently increased the oxidation of glucose, triggered the conversion of glucose to lactate, but decreased the formation of glutamate or glycogen from glucose and reduced the potential route for fatty acid synthesis and ribose synthesis [64]. The main novelty of that study was to discover the role of the recently described serine synthesis, one-carbon cycle, and glycine cleavage (SOGC) pathway in the fate of glucose carbons in the presence of added fructose in adipocytes. In this pathway, the glucose derived glycolytic metabolite 3-phosphoglycerate is used for the synthesis of serine, from which a fraction subsequently is converted to glycine in a reaction that is coupled with the one carbon metabolism pathway, yielding ATP [65]. The intermediates of the one carbon pathway generate NADPH, a key cofactor needed for fatty acid synthesis. Thus, the presence of fructose in adipocytes drives this alternate pathway, resulting in increased energy and CO_2_ production, which can be utilized in fructose-induced lipogenesis and fat storage in adipocytes [64].

A recent in vivo study supported the above described effects of fructose. Independent of whether fructose was provided either as a monosaccharide or in the form of sucrose combined with glucose, fructose increased the whole-body exogenous carbohydrate oxidation rate during prolonged exercise in volunteer healthy and trained cyclists [66]. A higher exogenous glucose oxidation rate was found to correlate with increased performance during prolonged high-intensity exercise [67] and co-ingestion of fructose further enhanced exogenous carbohydrate oxidation rates [66] and decreased gastrointestinal distress [68,69]. In type 1 diabetic individuals, the co-ingestion of glucose and fructose was also found to be beneficial during exercise compared to glucose alone. However, carbohydrate oxidation was lower but fat oxidation was higher upon co-ingestion of fructose and glucose compared to glucose alone in diabetic patients during exercise [70]. Additionally, the diabetic patients showed a glycogen-sparing effect in the working muscle, although their lactate production was elevated, as was also described previously in healthy individuals [71]. This suggests that the ingested fructose might be partially converted into lactate [72], or the fructose itself prompts conversion of glucose into lactate, as seen in vitro [64]. To conclude, these studies showed that co-ingestion of fructose with glucose may optimize fuel metabolism during exercise in healthy individuals by a more efficient energy supply due to higher carbohydrate oxidation and in diabetic patients by increased fat oxidation.

## 4. Effect of Fructose on Metabolic Disturbances

In the last couple of decades, the cellular and molecular mechanisms of adipocyte differentiation have been extensively studied and various hormones and growth factors affecting adipocyte differentiation in a positive or negative manner have been identified [9,73,74,75,76,77,78]. Although several clinical and in vitro studies defined the high lipogenicity of fructose and its stimulation of adipogenesis [19,79,80], further questions were raised on the exact mechanisms and regulatory factors involved behind this phenomenon. 

There is compelling evidence that oxidative stress is implicated in fructose-mediated adiposity, insulin resistance, and metabolic syndrome. In a study in rats, fructose induced the mRNA and protein expression of ER stress markers, including GRP-78, PERK, IRE1α, and CHOP in the liver [81]; which on one hand might contribute to the hepatic activation of SREBP-1c and lipid accumulation in fructose-induced NAFLD [38], and on the other hand the increased ER-stress is also suggested to cause hepatic insulin resistance by increasing de novo lipogenesis [10,82]. Another central role of the ER is to control the transport and metabolism of cholesterol, an essential component of cellular membranes, which is mainly regulated by transcription factors of the SREBP family [83]. 

Another aspect of the fructose-mediated metabolic effects, when it is rapidly metabolized in the liver, is its conversion to fructose-1-phosphate, which can cause intracellular phosphate depletion and AMP formation (Figure 1), resulting in the activation of AMP deaminase and the formation of uric acid [58]. Uric acid production has been identified as a sensitive measure of hepatic ATP depletion [84]. The serum uric acid can rapidly raise, up to a level of 2 mg/dL, after intravenous or oral fructose consumption [85,86]. Although this initial increase is transient, it was found that administration of fructose for several weeks increases fasting uric acid levels [87,88]. Elevated levels of uric acid have been associated with a series of pathological conditions, including insulin resistance, obesity, T2DM, and chronic kidney disease [57,89] and have been proposed as a risk factor for myocardial infarction and neurological diseases including stroke [90,91]. Lowering the uric acid level using the xanthine oxidase inhibitor febuxostat prevented the fructose-induced development of MetS [92]. One suggested mechanism includes the direct effect of uric acid on adipocytes. Using cultured adipocytes, evidence was provided for an induction of oxidative stress and inflammation by uric acid [93]. Knockdown of the xanthine oxidoreductase inhibited adipogenesis and PPARγ activity [94]. These studies implicate that uric acid might serve as an important regulator of adipogenesis; therefore, fructose-mediated uric acid formation might be associated with insulin resistance and MetS.

It is important to note that rats possess uricase, which degrades uric acid to allantoin, explaining why fructose does not increase the uric acid level effectively in this species. In experiments with rats, uricase inhibitors are needed; however, this leads to more than 10-fold increased uric acid levels in response to fructose administration [95]. In contrast, humans have no functional uricase due to an evolutionary mutation [96]. Another reason why the rat may be of limited relevance to study fructose toxicity is the fact that rats can produce ascorbate (vitamin C), which can block the adverse effects of fructose. As an antioxidant, vitamin C can attenuate uric acid-mediated vascular smooth muscle cell effects and hypertension [97,98]. 

In the adipose tissue, ER-stress induction also plays an important role in the pathomechanism of impaired differentiation processes [99]. During maturation, the ER environment of fibroblast-like preadipocytes must confront the demand of secreting enormous amounts of peptide and lipid mediators and storing energy in the form of TG in lipid droplets [100]. During nutrient overload and/or disturbances, cellular stress can lead to an impairment of ER function, limiting the capacity of proper protein folding and resulting in an accumulation of unfolded proteins in the ER lumen, ultimately leading to impaired adipocyte maturation. Such a mechanism for fructose-mediated ER-stress induction in adipocytes was reported recently by Marek et al. [101]. They provided important mechanistic insight into how fructose consumption not only influences ER redox status via depleting ERO-1α expression but also affects one of the key ER-stress signaling pathways by inducing XBP-1 splicing in the VAT of treated mice. Since the assembly and secretion of the beneficial anti-inflammatory and insulin-sensitizing adipokine adiponectin is regulated by ER chaperones such as ERO-1α and ERp44 [102], the fructose-mediated depletion of the biologically active high-molecular weight protein adiponectin might be explained by an altered ER homeostasis [101]. Furthermore, this study revealed macrophage infiltration and increased expression of inflammatory cytokines such as monocyte chemoattractant-1 (MCP-1) and tumor necrosis factor-α (TNF-α) in the VAT in response to fructose [101]. Importantly, all of these fructose-mediated effects were mediated by KHK. Although, the low-activity KHK (KHK-A) isoform is expressed in adipocytes [103] and alternative hexokinase-mediated fructose metabolism takes part in adipose tissue, it is more reasonable to imply that the observed fructose-mediated metabolic effects are triggered by KHK-C-dependent metabolism in the liver. These recent results suggest that the enhanced hepatic de novo lipogenesis and TG production affect adipose tissue via intermediary metabolic and inflammatory communication. The fructose-induced proinflammatory process with infiltrated macrophages in VAT and the caused adiponectin resistance are the main important contributors to insulin resistance and global metabolic changes in the situation of fructose over-consumption [101]. 

Regarding this hypothesis, an important role of the ER protein CHOP in modulating the polarity of adipose tissue macrophages was proposed recently [104]. A high fat diet (HFD; consisting of saturated fat, protein, and sucrose, i.e., 32% sunflower oil, 33% casein, 18% sucrose) resulting in the induction of ER-stress led to upregulation of CHOP expression in adipocytes, altering adipocyte function and suppressing microenvironment conditions, involving downregulation of Th2 cytokines needed to inhibit M2 polarization of macrophages infiltrated in the adipose. Hence ER-stress induction results in chronic inflammation in adipose tissue and insulin resistance at the whole-body level. In mice with CHOP deficiency, adipose tissue macrophage M2 polarization was maintained and these mice were protected against HFD-mediated metabolic effects and insulin resistance [104].

Another important mediator of adipogenesis and adipocyte function includes glucocorticoids. Although both the glucocorticoid receptor (GR) and the mineralocorticoid receptor (MR) are expressed in adipose tissue and have roles in regulating leptin expression, silencing experiments showed that GR has more important roles in mediating adipogenesis and adipokine production in human adipocytes [74,105]. In addition, the intracellular generation of active glucocorticoids in monocyte/macrophages regulates the release of pro-inflammatory molecules [106]. It was shown that the selective inhibition of 11β-HSD1 improved multiple MetS parameters, suppressed the inflammation of adipose tissue [107], exerted anti-inflammatory effects in lipopolysaccharide (LPS)-activated macrophages via the stimulation of heme oxygenase-1 [108], and reduced pro-inflammatory gene expression in atherosclerotic tissues [109] in rodent models. Fructose-induced proinflammatory effects in adipocytes or in macrophage infiltrating adipose tissue or the liver might be exacerbated by reduced GR signaling and/or enhanced MR signaling [105,110,111], or it may directly stimulate local glucocorticoid activation in these tissues (see below, Section 6). In this regard, the effects of glucocorticoids in the development and progression of T2DM and cardiovascular complications upon the excessive consumption of fructose-containing foods in our modern society need to be further investigated. In line with a role for oxidative stress-related complications, several studies implicated that the administration of antioxidants might prevent the fructose-induced adipose tissue dysfunctions [112], or the progression of steatosis and inflammation in NAFLD [113,114]. 

To conclude, excess lipid accumulation caused by chronic fructose over-feeding is known to be associated with ER-stress and cellular dysfunction in adipocytes. 

## 5. Role of 11β-HSD1 in Adipocyte Differentiation/Proliferation

A systemic glucocorticoid excess, as observed in Cushing’s disease, leads to obesity and all further symptoms of the MetS, with a pathological phenotype of dyslipidemia, insulin resistance, and hypertension [115,116]. However, in abdominally obese patients without Cushing’s disease, circulating cortisol levels are not elevated [115]. However, individuals with essential abdominal obesity have an impaired diurnal glucocorticoid rhythm with lower peak levels but higher levels during nadir [117,118]. The total excretion of urinary glucocorticoid metabolites is elevated, probably as a result of an increased hepatic clearance rate due to increased expression of 5α-reductase [21,119,120,121]. Since circulating glucocorticoid levels are in the normal range, this indicates a higher hypothalamus-pituitary-adrenal (HPA) axis activity. Importantly, the local cortisol synthesis in adipose tissue was found to be increased and is recognized as an important etiologic factor for obesity-related diseases [21,33,122]. Intracellularly, 11β-HSD1 is responsible for the generation of physiologically active glucocorticoids (cortisol, corticosterone) from their inert precursors (cortisone, 11-dehydrocorticosterone), thus regulating glucocorticoid access to glucocorticoid- and mineralocorticoid receptors [28,29,32,34]. It is known that 11β-HSD1 is elevated in adipose tissue in obesity [122,123], where it can contribute to metabolic complications. In contrast, 11β-HSD1 expression remained at normal levels or was found to be reduced in the liver in obesity and T2DM [123,124,125]. Investigations in transgene mice showed that a moderate overexpression of 11β-HSD1 in adipose tissue was sufficient to induce specific fat accumulation in the VAT [20]. These mice also presented with increased adipocyte size, especially in the VAT, as well as increased non-esterified fatty acid release. Conversely, transgenic 11β-HSD1 KO mice showed reduced hyperglycemia and VAT accumulation and improved insulin sensitivity compared to wild-type mice under conditions of stress and high-fat diet [126]. 

Earlier, our group reported the expression of hexose-6-phosphate dehydrogenase (H6PDH) in rat epididymal fat, as detected at the level of mRNA, protein, and activity [127]. Adipocytes are equipped with a functional glucose-6-phosphate transporter (G6PT)—H6PDH—11β-HSD1 system. As exemplified by the model compound metyrapone, an NADPH-depleting agent for modulating local glucocorticoid activation [128], all three components are potential pharmacological targets. Metyrapone administration caused a shift from 11β-HSD1 oxoreductase to dehydrogenase activity in both 3T3-L1-derived and human stem cell-derived differentiated adipocytes [128]. Furthermore, the depletion of luminal pyridine nucleotides in the ER attenuated 11β-HSD1 oxoreductase activity and the decreased accumulation of lipid droplets during preadipocyte differentiation.

During adipocyte maturation, at an early stage, the expression of 11β-HSD1 is low in pre-adipocytes, whereas it increases during the late phase. Earlier studies revealed that glucocorticoids play an important role in preadipocyte differentiation, as active glucocorticoids were required for terminal adipogenesis [129,130] and limit cell proliferation [131]. Inhibition of 11β-HSD1 activity by pharmacological agents or shRNA blocked the capability of inactive 11-oxoglucocorticoids to promote differentiation [132,133]. Thus, these observations emphasize the adipogenic role of glucocorticoids. 

## 6. Effect of Fructose on 11β-HSD1 Expression and Activity

**Figure 2:**
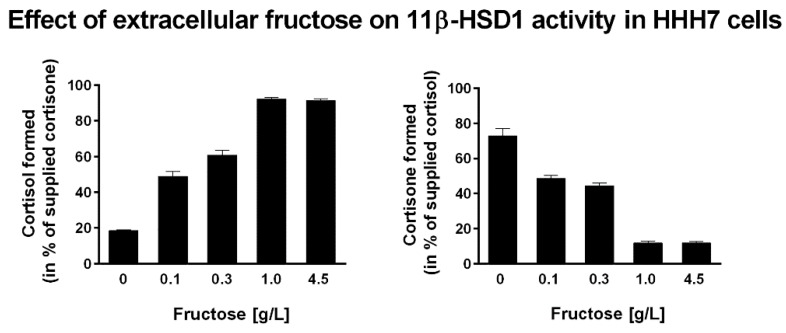
Exogenous fructose regulates 11β-HSD1 activity. Human embryonic kidney cells stably expressing human 11β-HSD1 and H6PDH (HHH7 cell clone [136]) were incubated with different fructose concentrations for 24 h, followed by determination of the 11β-HSD1 oxoreductase (left panel) and dehydrogenase (right panel) activities. Increasing concentrations of extracellular fructose shifted the activity from dehydrogenase to oxoreductase activity. Data represent mean ± S.D. from four independent experiments.

Recent evidence highlights a role of the ER as a nutrient sensor [134], supporting the cellular response to extreme nutritional conditions. The redox state of ER-luminal pyridine nucleotides determines the reaction direction of 11β-HSD1, and alterations of the redox state of pyridine nucleotides are well mirrored by cortisone reduction and cortisol oxidation capacity [29,135]. Over-nutrition with a high sugar load stimulates the local activation of glucocorticoids through the G6PT—H6PDH—11β-HSD1 triad. A previous study addressed the effect of extracellular glucose availability on 11β-HSD1 activity [135]. Lowering glucose concentration in the culture medium caused a decrease in the NADPH/NADP^+^ ratio, which consequently resulted in a shift from 11β-HSD1 oxoreductase to dehydrogenase activity, thereby lowering the cortisol/cortisone ratio. As reported earlier, at 1 g/L of glucose, 11β-HSD1 oxoreductase activity decreased by 40% compared to cells kept in 4.5 g/L glucose medium. To see whether fructose might have a similar effect, we measured 11β-HSD1 oxoreductase and dehydrogenase activities in cells stably coexpressing 11β-HSD1 and H6PDH (HHH7 clone [136]) with different fructose concentrations in the culture medium (Figure 2). Interestingly, in contrast to glucose, the presence of 1 g/L fructose in the medium as the only carbohydrate source was still capable of maintaining high oxoreductase activity, indicating a high intraluminal NADPH/NADP^+^ ratio [35]. To extend this dose-dependent effect, we incubated HHH7 cells with various concentrations of fructose. The results showed that even at fructose concentrations as low as 0.1 g/L, efficient 11β-HSD1 oxoreductase activity was observed (50% at 0.1 g/L compared to 4.5 g/L). This suggests that fructose constitutes a more efficient source of ER-luminal NADPH than glucose. 

A possible explanation for the fact that fructose seems to be a preferred source for ER-luminal NADPH generation and therefore stimulation of 11β-HSD1-dependent glucocorticoid activation, compared to glucose, might be provided by its intracellular metabolism. Fructose is metabolized in the liver to fructose-1-phosphate, bypassing the key glycolysis regulatory enzyme phosphofructokinase, leading to enhanced lipogenesis. The adipose does not express fructokinase and fructose is converted by hexokinase to F6P [137]. An in vitro study using rat and porcine liver microsomes found that G6P and F6P but not galactose-1-phosphate, glucose-1-phosphate, and fructose-1-phosphate stimulated 11β-HSD1 oxoreductase activity [24]. Interestingly, F6P, unlike G6P, failed to increase 11β-HSD1 oxoreductase activity in porcine adipose microsomes, and the reason for this observation remains unclear. Later, another study using rat liver and adipose microsomes demonstrated that F6P efficiently induced 11β-HSD1 oxoreductase activity. This study also provided evidence for the existence of a F6P transporter in the ER membrane that is distinct of the G6P transporter G6PT, and for the existence of an ER-luminal F6P isomerase, which forms G6P for H6PDH-dependent NADPH generation [25]. Importantly, F6P did not directly serve as a substrate of H6PDH but needed to be first converted to G6P. The luminal F6P isomerase showed different properties than its cytoplasmic counterpart, suggesting that the ER-luminal enzyme is encoded by a different gene. Identification of the gene encoding this ER-luminal F6P isomerase as well as that for the 6-phosphogluconate dehydrogenase (which generates another NADPH molecule in addition to H6PDH) will be important for a better understanding of the coupling of energy status and 11β-HSD1-mediated glucocorticoid activation. 

An alternative explanation for the superiority of fructose in ER-luminal NADPH generation can be the preferential transport of fructose and F6P over glucose and G6P through the plasma membrane and ER membrane, respectively. Fructose stimulates its own uptake via GLUT5 at the gene expression level (see above), while the most important glucose transporter in adipocytes, GLUT4, is active only under hyperglycemic conditions. The rates of F6P and G6P transport through the ER-membrane have not yet been compared, an issue that needs to be addressed in future research.

Fructose not only stimulates 11β-HSD1 oxoreductase activity by increasing luminal NADPH generation but also by affecting gene expression. An increased 11β-HSD1 expression and activity was observed in mouse 3T3-L1 adipocytes that were cultivated in medium containing fructose as the only carbohydrate source instead of glucose [35]. As a possible explanation for the elevated expression, an increased ratio of the transcription factors C/EBPα to C/EBPβ, reported earlier to be involved in the transcriptional regulation of 11β-HSD1 [138,139], was detected. Moreover, 3T3-L1 adipocytes differentiated in fructose containing medium had elevated expression of GLUT5, thus further enhancing fructose uptake and stimulating 11β-HSD1 expression and activity. Additionally, lipolysis was induced with increased phosphorylation of perilipin, enhanced expression of hormone sensitive lipase and adipocyte triglyceride lipase, and elevated release of glycerol and FFA. This suggested fructose as a potent adipocyte differentiation stimulant via increasing local glucocorticoid activation. 

The above described observations were supported by animal experimentation. A very recent in vivo experiment with Sprague Dawley rats fed with a fructose solution (10% (*w*/*v*)) for 9 weeks confirmed our (above established) hypothesis, that fructose overload promotes glucocorticoid production through the enhanced expression and activity of 11β-HSD1 and H6PDH, supplying further NADPH in rat epididymal white adipose tissue [140]. Importantly, these rats developed some of the characteristic features of MetS, such as hypertriglyceridemia and hypertension. Other investigators showed that a shorter exposure of 24 h to a high fructose diet in rats resulted in an elevated expression of 11β-HSD1 in the liver and in VAT [23]. An enhanced expression of 11β-HSD1 as well as GR-regulated lipogenic genes accompanied by an induced adipogenesis was suggested by studies using Wistar rats on 10% fructose in the drinking water for a 9 week period [141,142,143]. Male rats that were subjected to both fructose-rich diet and chronic unpredictable stress had slightly elevated corticosterone levels, higher 11β-HSD1 expression (but not H6PDH, contrary with the previous animal study), and evidence for increased GR activation [142]. Also, acetyl-CoA-carboxylase, fatty acid synthase and hormone sensitive lipase expression levels were elevated. This may suggest that chronic stress further exacerbates the fructose-mediated induction of 11β-HSD1 and local glucocorticoid effects on lipolysis and adipogenesis. A recent study with mice that were fed a high-fructose diet for 60 days found elevated glucocorticoid levels in liver and adipose tissue as well as enhanced GR in the nucleus and activation of its target genes [144]. Plasma FFA, TG, insulin, and glucose were increased while hepatic glycogen was decreased. Treatment with the GR antagonist RU486 lowered plasma lipids, tissue glucocorticoids, and GR activation, as well as the expression of its target genes. Additionally, lipid accumulation in adipose tissue decreased and insulin sensitivity was improved. Interestingly, the high-fructose diet resulted in an increased expression of 11β-HSD1, H6PDH, and G6PT in the liver and adipose. Furthermore, the anti-lipogenic transcription factor Hes-1 was down regulated by elevated GR activity while the expression of PPARγ, CD36, and SREBP1-c were enhanced, explaining the elevated FFA and TG production. These observations suggest that high fructose consumption leads to elevated expression and activity of the 11β-HSD1-H6PDH-G6PT triad, promoting local GR activation and glucocorticoid-mediated stimulation of lipolysis and adipogenesis. It will be important to see in follow-on studies whether the selective inhibition of 11β-HSD1 may protect from the adverse metabolic effects of high-fructose consumption. 

In this regard, a 16 week treatment of male mice with the American Lifestyle-induced Obesity Syndrome (ALIOS) diet (ad libitum feeding of 45% calories from fat, 11.6% transfats, and 42 g/L high fructose corn syrup (55% fructose, 45% glucose) in the drinking water) recapitulated obesity, insulin resistance, dyslipidemia, and the spectrum of nonalcoholic fatty liver disease (NAFLD) [145]. However, global 11β-HSD1 KO mice were not protected from the metabolic dysregulation following the 16 week ALIOS diet. Glucocorticoids are known to promote steatosis, among other mechanisms by stimulating lipolysis within the adipose tissue, and this leads to increased FFA delivery to the liver, for the production of lipids through increased hepatic de novo lipogenesis [146,147,148]. 11β-HSD1 KO mice were protected from steatohepatitis upon adding glucocorticoids in the drinking water but as a standard rodent chow [149]. This study also emphasizes the importance of adipose 11β-HSD1 and its impact on the hepatic phenotype. Interestingly, the ALIOS diet led to an early transition to hepatic inflammatory disease with elevated markers of inflammation, immune cell infiltration, and fibrosis in 11β-HSD1 KO mice, indicating a transition to non-alcoholic steatohepatitis (NASH) [145]. Why the global 11β-HSD1 KO mice were not protected against the ALIOS diet in the study by Larner et al. [145], but 11β-HSD1 KO mice were resistant against hyperglycemia induced by obesity or stress in the study by Kotelevtsev et al. [126], remains unclear. Analysis of the differences in animal maintenance, diet, and treatment duration may provide an explanation. It needs to be noted that the life-long adaptation by compensatory mechanisms including an elevated adrenal glucocorticoid production may be responsible for the lack of protection from the high fructose, high transfat diet in 11β-HSD1 KO mice, and that the administration of pharmacological inhibitors, especially when targeted to the adipose tissue, may lead to a different outcome. 

## 7. Conclusions

**Figure 3:**
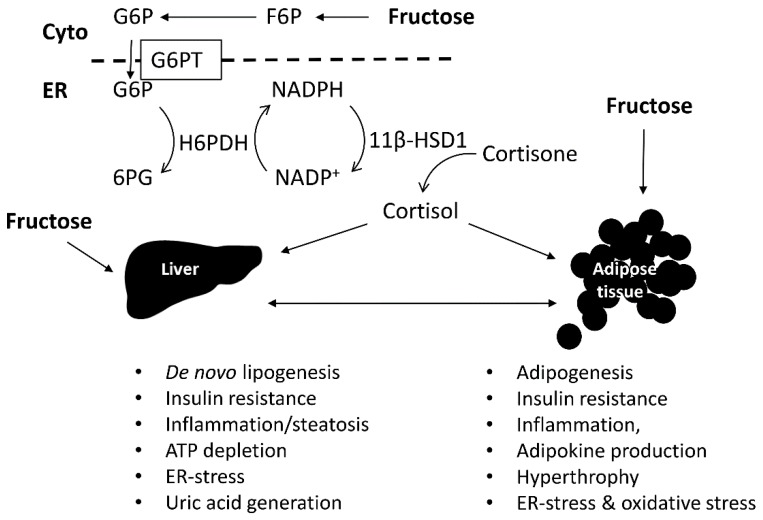
Effect of fructose on liver and adipose tissue, their interconnections, and impact of glucocorticoid activation. Excessive fructose consumption is thought to be associated with hepatic steatosis, cellular stress, and inflammation of the MetS. The enhanced glucocorticoid production also has a crucial role in the regulation of adipocyte differentiation and cellular metabolism. 11β-HSD1, 11β-hydroxysteroid dehydrogenase 1; 6PG, 6-phosphogluconate; Cyto, cytoplasm; ER, endoplasmic reticulum; F6P, fructose-6-phosphate; G6P, glucose-6-phosphate; G6PT, glucose-6-phosphate transporter in the ER-membrane; H6PDH, hexose-6-phosphate dehydrogenase

Both excessive fructose consumption and increased intracellular glucocorticoid activation have been suggested to contribute to the pathogenesis of the MetS (Figure 3). Fructose is suggested to be the most hypertriglyceridemic sugar. However, it is important to investigate whether abdominal obesity exacerbates the hypertriglyceridemic effect of the high fructose diet and whether increased glucocorticoids further aggravate the adverse metabolic effects of high fructose. Independently of the consumed fructose, elevated glucocorticoids and central obesity, especially visceral obesity, are associated with a higher risk for T2DM and MetS. Therefore, future investigations on the effects of fructose should consider the source and dietary form of fructose (solid food or beverage) and should include careful controls regarding the sex-, genetics-, stress-, and obesity-related differences in responsiveness. 

The importance of the G6PT-H6PDH-11β-HSD1 system in the ER-lumen received a distinct focus in the past few years, providing a novel pharmaceutical potential to intervene in the progression of MetS and prevent its diabetic and cardiovascular consequences. However, there are important questions remaining. Whether or not pharmacological inhibition of H6PDH or G6PT may offer therapeutic benefits remains fully unexplored and further basic research to better understand the functions of these two proteins is needed. The fact that global 11β-HSD1 KO mice, which are subjected to adaptation by life-long compensatory mechanisms, were not protected against the ALIOS diet in the study by Larner et al. [145] is an argument against protection from adverse metabolic effects of high fructose containing diet by the pharmacological inhibition of 11β-HSD1. However, selective inhibition of 11β-HSD1 in adipose tissue might be superior to global enzyme deficiency due to more pronounced feedback regulation and increased adrenal glucocorticoid production in the latter situation. Thus, the effect of selective inhibition of 11β-HSD1 specifically in adipose tissue should be investigated. Furthermore, species-specific differences need to be considered. Fructose as well as glucocorticoid metabolism in rodents and human are different in several aspects, and, ideally, clinical studies should be performed to better understand the link between high fructose intake and glucocorticoid action. 

## Abbreviations

AMPK: Adenosine 5′-monophosphate (AMP)-activated protein kinase; ATP: Adenosine Triphosphate; CVD: Cardiovascular Disease; CHOP: CCAAT-enhancer-binding protein homologous protein; ER: endoplasmic reticulum; F1P: fructose-1-phosphate; G6P: glucose-6-phosphate; G6PT: G6P translocase; GLUT5, glucose transporter 5; Fru-1-P: Fructose-1-phophate; FFA: free fatty acid; H6PD: hexose-6-phosphate dehydrogenase; HFD: high fat diet; 11β-HSD1: 11β-hydroxysteroid dehydrogenase type 1; MetS: metabolic syndrome; NADPH: reduced nicotinamide adenine dinucleotide phosphate; NAFLD: nonalcoholic fatty liver disease; NASH: nonalcoholic steatohepatitis; PPARα: Peroxisome Proliferator-Activated Receptor α; ROS: reactive oxygen species; SREBP1c: Sterol Response Element Binding Protein 1c; T2DM: type II diabetes mellitus; TG: triglycerides; TNF: tumor necrosis factor; UPR: unfolded protein response; VAT: visceral adipose tissue; VLDL: Very Low Density Lipoprotein; XBP-1: X-box binding protein 1.
